# Impact of fixed appliance treatment on root resorption in root canal-treated teeth: a systematic review and meta-analysis

**DOI:** 10.2340/aos.v84.43642

**Published:** 2025-05-20

**Authors:** Yongfang Feng, Rong Wang, Yumin Zhou, Shengnan Zhan

**Affiliations:** a Department of Dentistry and Endodontics, Shaoxing Stomatological Hospital, Shaoxing City, China; b Department of Oral Prevention, Shaoxing Stomatological Hospital, Shaoxing City, China

**Keywords:** root resorption, orthodontic treatment, root canal treatment, fixed appliance, meta-analysis

## Abstract

**Objective:**

The relationship between orthodontic treatment and root resorption in endodontically treated teeth remains controversial. This systematic review and meta-analysis aimed to evaluate the effects of fixed appliance treatment on root resorption in root canal-treated teeth compared to vital teeth.

**Methods:**

A comprehensive literature search was conducted across multiple databases including PubMed, Embase, Cochrane Library, Web of Science, Sinomed, CNKI, and Wanfang. Studies comparing root resorption between root canal-treated teeth and vital teeth during orthodontic treatment were included. The methodological quality was assessed using the Cochrane risk of bias tool. Standardized mean differences (SMD) with 95% confidence intervals (CI) were calculated using random-effects models.

**Results:**

Ten studies involving 266 patients met the inclusion criteria. The overall effect showed no significant difference in root resorption between root canal-treated teeth and vital teeth (SMD = -0.08, 95% CI: -0.24 to 0.08, *I*² = 7.0%). Subgroup analyses revealed no significant differences based on measurement methods, extraction versus non-extraction treatment, or tooth position. However, extraction cases demonstrated a numerical trend toward greater root resorption in root canal-treated teeth (SMD = -0.70, 95% CI: -1.50 to 0.11, *I*² = 67.1%, *p* = 0.048), while the non-extraction group showed no meaningful differences (SMD = -0.07, 95% CI: -0.42 to 0.28, *I*² = 49.2%, *p* = 0.096).

**Conclusions:**

This meta-analysis suggests that root canal-treated teeth do not show significantly different root resorption patterns compared to vital teeth during orthodontic treatment. However, extraction cases may require careful monitoring due to a tendency toward increased root resorption.

## Introduction

Orthodontic treatment has emerged as a cornerstone in addressing malocclusion, optimizing occlusal relationships, and enhancing facial aesthetics. Among various orthodontic modalities, fixed appliance therapy has become a preferred choice due to its precision and predictability in tooth movement [1]. Fixed appliances, comprising brackets, archwires, and elastics, apply controlled mechanical forces to guide teeth through alveolar bone remodeling. Despite its clinical success, orthodontic treatment is not without risks, with root resorption standing out as one of the most significant and irreversible complications [2]. Root resorption involves the progressive loss of dental root structure, leading to root shortening, surface defects, and, in severe cases, tooth mobility or loss, ultimately compromising oral health and patient quality of life [3].

The etiology of root resorption is multifactorial, arising from complex interactions between biological responses and clinical variables. Orthodontic forces, essential for tooth movement, induce localized inflammation and osteoclast activation, which may inadvertently trigger root resorption [4].

Among the numerous factors affecting root resorption, pulp status, especially the health of the pulp, is considered one of the key factors. Root canal therapy, as a common method for treating pulp diseases, involves the removal of infected pulp tissue and the filling of sealing materials to restore tooth function and preserve tooth structure [5]. However, root canal treatment leads to the tooth losing vitality, and the reactivity of periodontal tissues may decrease, thereby affecting the adaptability of the tooth under orthodontic force. Specifically, teeth after root canal treatment, due to the loss of nerve and blood supply, have reduced perception and reactivity to external stimuli, which may lead to decreased tolerance of the root to orthodontic force, increasing the risk of root resorption. In details [6], root canal-treated teeth show significant differences in biomechanical responses compared to vital teeth due to the loss of pulp vitality. The loss of the pulp neurovascular system interrupts stress signaling pathways mediated by mech-anoreceptors in dentinal tubules, weakening the periodontal ligament’s dynamic regulation ability. Additionally, the removal of pulp tissue eliminates the reparative capacity of odontoblasts and pulp stem cells, which can secrete reparative dentin and modulate inflammatory factors to mitigate root resorption damage. Furthermore, the atrophy or fibrosis of periapical blood vessels reduces blood supply to the periodontal ligament, inhibiting the dynamic balance between osteoblasts and osteoclasts and diminishing the tooth root’s biological resistance to resorption. Collectively, these mechanisms lead to decreased adaptability of root canal-treated teeth to orthodontic forces and potentially increase the risk of root resorption. However, existing evidence remains controversial, emphasizing the need for systematic integration of research findings.

The methods used to assess root resorption in the included studies may influence the results of our meta-analysis. Traditional two-dimensional imaging methods, such as periapical radiographs and panoramic radiographs, are widely used in clinical practice due to their low cost and ease of operation. However, these methods have limitations in accurately detecting root resorption, potentially leading to underestimation of its extent. In contrast, Cone-Beam Computed Tomography (CBCT) provides three-dimensional imaging, which can more accurately and comprehensively assess the degree and distribution of root resorption. However, its high cost and radiation dose limit its widespread use in clinical practice. The differences in imaging methods used across studies may introduce heterogeneity in the reported results, affecting the overall conclusions of our meta-analysis [7–9].

In addition, whether to extract teeth or not during orthodontic treatment is also an important factor influencing root resorption. Tooth extraction may alter the distribution of orthodontic force, affecting the stress state on teeth and thereby influencing the degree of root resorption [10]. Some studies indicate that after extraction, due to the increased space between teeth, the redistribution of orthodontic force may cause certain teeth to bear greater movement force, thereby increasing the risk of root resorption [11]. However, other studies suggest that whether to extract or not does not significantly affect root resorption, and it depends more on the specific treatment plan and force application methods [12].

The position of the teeth is also an important factor affecting root resorption. Anterior and posterior teeth experience differences in the direction and magnitude of orthodontic forces during treatment, leading to different patterns of root resorption in the orthodontic process [13]. For example, anterior teeth typically require greater rotation and movement, while posterior teeth involve more vertical and horizontal movements, which may lead to differences in root resorption between anterior and posterior teeth [14]. Moreover, different types of teeth, such as incisors, canines, and molars, have different root structures and morphologies, which may influence their susceptibility to root resorption [15]. Despite the extensive research on root resorption during orthodontic treatment, systematic reviews and meta-analyses specifically focusing on root resorption in endodontically treated teeth during orthodontic therapy remain limited. While individual studies have provided numerous insights into this issue, comprehensive analyses integrating results from multiple studies are crucial for a deeper understanding. Existing reviews typically cover broad aspects of orthodontic-induced root resorption, but detailed investigations into the specific impact of root canal treatment on root resorption during orthodontic treatment, particularly in terms of long-term outcomes, specific force magnitudes, and the influence of different treatment protocols (e.g. extraction vs. non-extraction), are still insufficient.

This study aims to fill these gaps by conducting a systematic review and meta-analysis to assess the impact of fixed appliance treatment on root resorption in root canal-treated teeth. By focusing on long-term outcomes, specific force application techniques, and the influence of different treatment protocols, we aim to provide a comprehensive evaluation of the current evidence and highlight areas that require further investigation. This will help clarify the true impact of root canal treatment on root resorption during orthodontic treatment and provide a scientific basis for clinical practice, assisting orthodontists in better assessing the risk of root resorption when formulating treatment plans.

## Methods

### Search strategy

A comprehensive literature search was conducted across multiple electronic databases to identify relevant studies examining the relationship between fixed appliance treatment and root resorption in root canal-treated teeth. The search encompassed PubMed, Embase, Cochrane Library, Web of Science, Sinomed, CNKI, and Wanfang databases. The search strategy employed a combination of Medical Subject Headings (MeSH) terms and free-text keywords, including ‘Fixed Appliance Treatment’, ‘Root Resorption’, ‘Root Shortening’, ‘Root Canal Therapy’, and ‘Nonvital Teeth’.

Exact Search Terms and Boolean Operators:

PubMed:

(Fixed Appliance Treatment OR Orthodontic Brackets OR Archwires) AND (Root Resorption OR Root Shortening) AND (Root Canal Therapy OR Endodontic Treatment OR Nonvital Teeth)

Date Range: 1980–2024

Embase:

(Fixed Appliance Treatment OR Orthodontic Brackets OR Archwires) AND (Root Resorption OR Root Shortening) AND (Root Canal Therapy OR Endodontic Treatment OR Nonvital Teeth)

Date Range: 1980–2024

Cochrane Library:

(Fixed Appliance Treatment OR Orthodontic Brackets OR Archwires) AND (Root Resorption OR Root Shortening) AND (Root Canal Therapy OR Endodontic Treatment OR Nonvital Teeth)

Date Range: 1980–2024

Web of Science:

(Fixed Appliance Treatment OR Orthodontic Brackets OR Archwires) AND (Root Resorption OR Root Shortening) AND (Root Canal Therapy OR Endodontic Treatment OR Nonvital Teeth)

Date Range: 1980–2024

Sinomed:

(Fixed orthodontic treatment OR Brackets OR Archwire) AND (Root resorption OR Root shortening) AND (Root canal treatment OR Pulp treatment OR Non-vital tooth)

Date Range: 1980–2024

CNKI:

(Fixed orthodontic treatment OR Brackets OR Archwire) AND (Root resorption OR Root shortening) AND (Root canal treatment OR Pulp treatment OR Non-vital tooth)

Date Range: 1980–2024

Wanfang:

(Fixed orthodontic treatment OR Brackets OR Archwire) AND (Root resorption OR Root shortening) AND (Root canal treatment OR Pulp treatment OR Non-vital tooth)

Date Range: 1980–2024

This extensive search yielded an initial total of 162 articles, with 41 from PubMed, 82 from Embase, 25 from Cochrane, 9 from Web of Science, 1 from Sinomed, 4 from CNKI, and 68 from Wanfang.

### Selection criteria

The inclusion criteria for this meta-analysis were carefully defined to ensure the selection of relevant and high-quality studies. Eligible studies were required to be clinical trials that compared root resorption between root canal-treated teeth and vital teeth during orthodontic treatment. The study population consisted of patients receiving fixed orthodontic appliance treatment, with interventions specifically focused on orthodontic treatment of root canal-treated te eth. The control group comprised vital teeth undergoing orthodontic treatment in the same patients or comparable patient groups. All included studies needed to provide quantitative assessments of root resorption using established radiographic methods.

### Study selection

The study selection process followed a systematic and rigorous approach in accordance with established systematic review guidelines. After the initial database search, duplicate articles were removed, resulting in 114 unique studies for screening. The selection process proceeded through multiple stages of evaluation. Initial title screening led to the exclusion of 67 articles that did not meet the basic criteria. Subsequently, abstract review resulted in the elimination of an additional 35 articles that did not align with the study objectives or methodology requirements. The remaining 12 articles underwent full-text assessment, during which 2 more articles were excluded due to methodological limitations or insufficient data reporting. This thorough selection process ultimately yielded 10 studies that met all inclusion criteria and were suitable for the meta-analysis.

### Data extraction

Data extraction was performed independently by two reviewers to ensure accuracy and completeness. The extracted information encompassed various aspects of each study, including study characteristics such as author names, publication year, and country of origin. Patient demographic data including gender distribution and age ranges were recorded. Treatment parameters were documented, including whether the orthodontic treatment involved extraction or non-extraction approaches, as well as the duration of treatment. Information about tooth position and the specific methods used for measuring root resorption was also collected. In cases where the two reviewers disagreed on data extraction, a third reviewer was consulted to reach consensus through discussion.

### Quality assessment

The methodological quality of the included studies was evaluated using the Cochrane risk of bias tool, which provides a comprehensive framework for assessing potential sources of bias in clinical trials. This assessment examined multiple domains including the methods of random sequence generation and allocation concealment in the study design. The implementation of blinding procedures for both participants and personnel was evaluated, as well as the blinding of outcome assessment. The completeness of outcome data and potential selective reporting were carefully scrutinized. Additional sources of potential bias were also considered in the overall quality assessment.

### Statistical analysis

The meta-analysis employed standardized mean differences (SMD) with 95% confidence intervals (CI) as the primary effect measure. Heterogeneity among studies was assessed using both I² statistics and chi-square tests, with significant heterogeneity defined as *I*² > 50% or *p* < 0.1. In cases where significant heterogeneity was detected, random-effects models were applied to account for the variation between studies. The analysis included several subgroup analyses to examine potential sources of heterogeneity and to investigate specific aspects of treatment outcomes. These analyses considered different measurement methods used across studies, compared extraction versus non-extraction treatment approaches, evaluated differences between anterior and posterior tooth positions, and examined the impact of treatment duration. All statistical analyses were performed using appropriate meta-analysis software, with statistical significance determined at *p* < 0.05.

## Results

### Study characteristics

**Figure 1:**
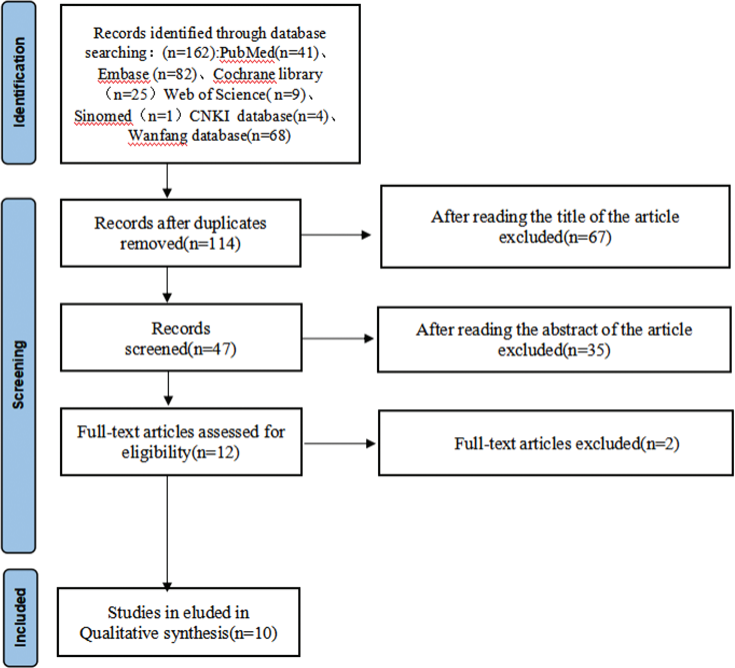
Study selection flow diagram.

Through systematic literature screening (Figure 1), 10 studies published between 1995 and 2018 were included in the final analysis. The characteristics of included studies are summarized in Table 1 [16–25], involving a total of 266 patients. The sample size across studies ranged from 6 to 68 patients, with ages ranging from 11 to 38 years. Gender distribution was reported in eight studies, with a total of 103 males and 163 females. Treatment duration varied across studies, ranging from 1 year to 3.17 years, with a mean duration of approximately 2 years. The included studies utilized various imaging modalities for assessing root resorption: three studies employed CBCT, four used panoramic radiographs, and three used periapical radiographs.

**Table 1:** Basic characteristics of included studies.

Study included	Gender		Age	Tooth position	Orthodontic treatment		Treatment duration	The subject of study		Measurement method
	Male	Female	Extracted		Non-extracted	Study group	Control group			
Castro 2015 [16]	2	4	11–15	Premolars and Molars	0	6	22 months	Root Canal Treatment	Same Side Vital Pulps	Cone Beam CT
Esteves 2007 [17]	-	-	-	Upper Central Incisors	-	-	>20 months	(Root Canal Treatment >1 year) Root Canal Treatment	Same Side Vital Pulps	Periapical Radiographs
Khan 2018 [18]	17	13	26.37 ± 2.40	Permanent Teeth	12	18	(3.17 ± 1.09 years)	(Root Canal Treatment >1 year) Root Canal Treatment	Same Side Vital Pulps	Panoramic Radiographs
Lee 2016 [19]	8	27	25.23 ± 4.90	Permanent Teeth	15	20	(26.58 ± 8.58 months)	(Root Canal Treatment >1 year) Root Canal Treatment	Same Side Vital Pulps	Panoramic Radiographs
Llamas-Carreras 2012 [20]	14	24	30.7 ± 10.2	Upper Incisors	-	-	(24.0 ± 12.0 months)	Root Canal Treatment	Same Side Vital Pulps	Panoramic Radiographs
Mirabella 1995 [21]	-	-	34.5 ± 9.0	Upper Front Teeth	-	-	2.07 ± 0.70 years	Root Canal Treatment	Same Side Vital Pulps	Periapical Radiographs
Ni M 2016 [22]	6	10	20.3 ± 3.2	Upper Front Teeth	0	16	(1.5 ± 0.2 years)	Root Canal Treatment	Same Side Vital Pulps	Cone Beam CT
Yang 2007 [23]	0	13	13–17	Upper Incisors	13	0	Average 1.7 years	(Root Canal Treatment >3 months) Root Canal Treatment	Same Side Vital Pulps	Periapical Radiographs
Wang 2017 [24]	30	30	12–38	Permanent Teeth	-	-	1–1.5 years	Root Canal Treatment	Same Side Vital Pulps	Panoramic Radiographs
Han 2018 [25]	26	42	23.4 ± 3.1	Upper Front Teeth	0	68	(1.5 ± 0.2 years)	Root Canal Treatment	Same Side Vital Pulps	Cone Beam CT

CT: Computed Tomography.

### Quality assessment outcomes

**Figure 2:**
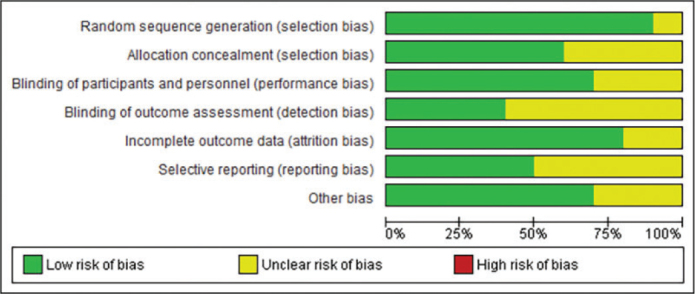
Risk of bias graph.

**Figure 3:**
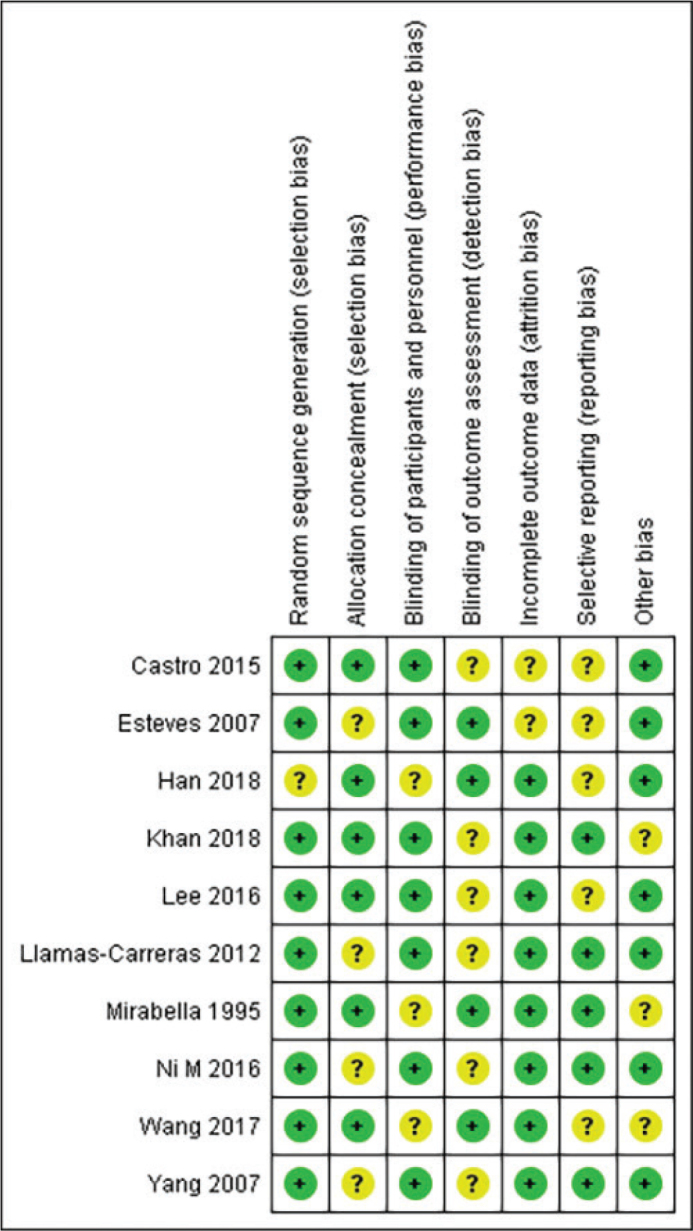
Risk of bias summary.

The methodological quality assessment revealed generally moderate to good quality across the included studies (Figures 2 and 3). Most studies demonstrated low risk of bias in random sequence generation and blinding procedures. The implementation of blinding was particularly well executed across studies, contributing to the reliability of the findings. However, some studies showed unclear risk in allocation concealment and incomplete outcome reporting.

### Primary analysis results

**Figure 4:**
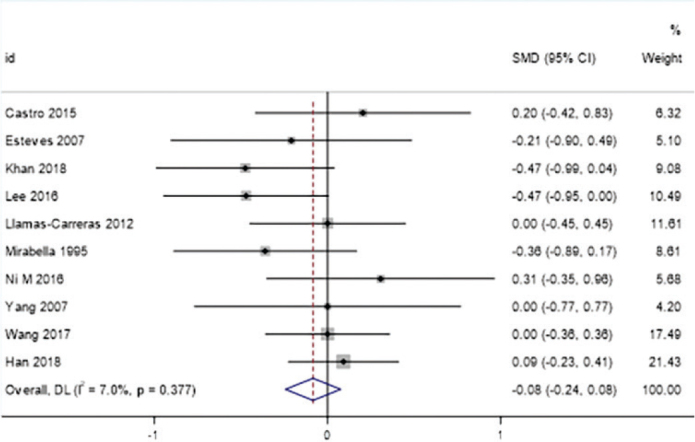
Forest plot of overall effect.

The meta-analysis showed no significant difference in the degree of root resorption between root canal-treated teeth and vital teeth during orthodontic treatment (SMD = -0.08, 95% CI: -0.24 to 0.08). The heterogeneity between studies was low (*I*² = 7.0%, *p* = 0.377), indicating consistency in the results (Figure 4).

### Subgroup analysis by measurement method

**Figure 5:**
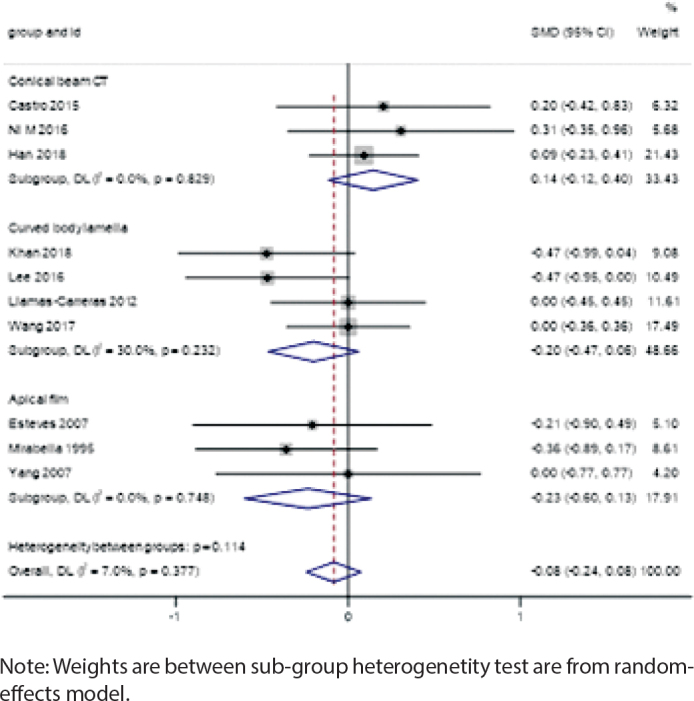
Forest plot by measurement method.

Measurement Method: Studies based on CBCT showed slightly higher root resorption in root canal-treated teeth (SMD = 0.14, 95% CI: -0.12 to 0.40), while periapical radiographs indicated reduced resorption (SMD = -0.23, 95% CI: -0.60 to 0.13). The panoramic radiograph group showed no significant difference (SMD = -0.20, 95% CI: -0.47 to 0.06) (Figure 5).

### Impact of extraction vs. non-extraction treatment

**Figure 6:**
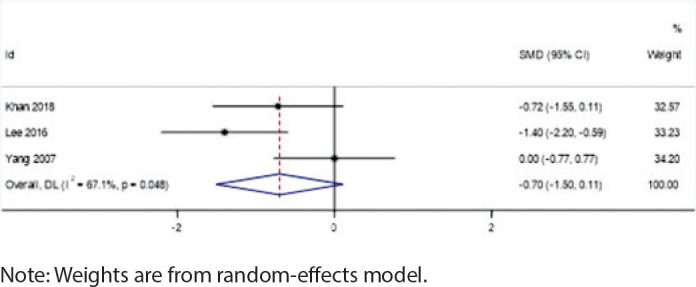
Forest plot of extraction cases.

**Figure 7:**
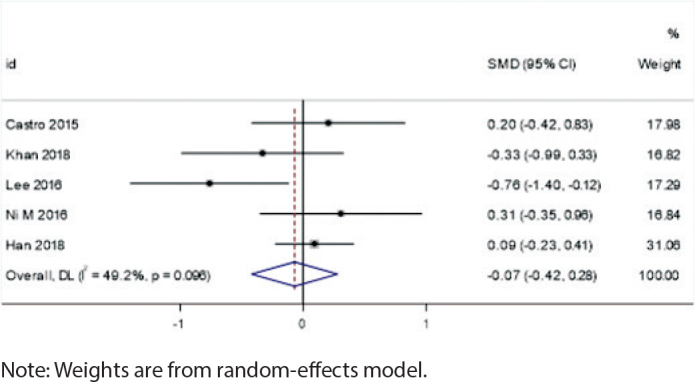
Forest plot of non-extraction cases.

Extraction cases showed a potential increase in root resorption risk for root canal-treated teeth (SMD = -0.70, 95% CI: -1.50 to 0.11, *I*² = 67.1%, *p* = 0.048), although this difference did not reach statistical significance. This suggests that clinicians should consider closer monitoring of root resorption in these cases. In contrast, the non-extraction group showed negligible differences in root resorption between root canal-treated teeth and vital teeth (SMD = -0.07, 95% CI: -0.42 to 0.28, *I*² = 49.2%, *p* = 0.096), indicating that root canal treatment alone may not significantly affect root resorption risk in this context (Figures 6 and 7).

### Tooth position analysis

**Figure 8:**
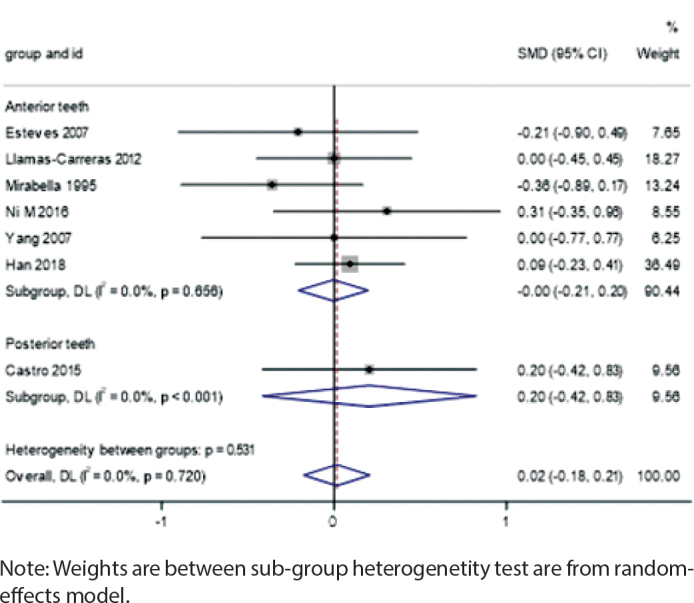
Forest plot by tooth position.

In the posterior teeth group, root canal-treated teeth showed a slight but non-significant increase in root resorption (SMD = 0.20, 95% CI: -0.42 to 0.83, *I*² = 0.0%, *p* < 0.001), while no differences were observed in the anterior teeth group (SMD = -0.00, 95% CI: -0.21 to 0.20, *I*² = 0.0%, *p* = 0.656) (Figure 8).

## Discussion

This systematic review and meta-analysis aim to explore the impact of fixed appliance treatment on root resorption in teeth that have undergone root canal therapy and to comprehensively analyze the roles of various clinical factors in this process. Through the combined analysis of 10 included studies, we found that overall, teeth that have undergone root canal therapy did not significantly increase the risk of root resorption during orthodontic treatment (SMD = -0.08, 95% CI: -0.24 to 0.08). This conclusion is consistent with some previous studies, supporting the notion that root canal therapy itself may not be an independent risk factor for root resorption [26].

The *I*² statistic is a crucial measure of heterogeneity among the included studies, reflecting the degree of inconsistency in the results. In our meta-analysis, the overall *I*² value for the comparison of root resorption between root canal-treated teeth and vital teeth was 7.0% (*p* = 0.377), indicating low heterogeneity. This suggests that the results across the studies were relatively consistent, which may be attributed to the similar study designs, patient populations, and measurement methods employed [27].

However, subgroup analyses revealed varying degrees of heterogeneity based on different factors:

### Measurement methods

- The CBCT subgroup showed no heterogeneity (*I*² = 0%), indicating high consistency in the results obtained using this advanced imaging technique. CBCT provides detailed three-dimensional imaging, which allows for more accurate detection of root resorption.
- Similarly, the panoramic radiograph subgroup (*I*² = 0%) and periapical radiograph subgroup (*I*²= 0%) also showed no heterogeneity. These traditional two-dimensional imaging methods, despite their limitations, provided consistent results across the studies.

### Extraction vs. non-extraction treatment

- The extraction subgroup exhibited significant heterogeneity (*I*² = 67.1%, *p* = 0.048), suggesting that the effect of orthodontic treatment on root resorption in root canal-treated teeth varied considerably among studies involving extraction. This high heterogeneity may be related to the redistribution of orthodontic forces after extraction, leading to increased root resorption risk in certain teeth.
- In contrast, the non-extraction subgroup showed moderate heterogeneity (*I*² = 49.2%, *p* = 0.096), indicating that the effect of orthodontic treatment on root resorption was more consistent in studies without extraction.

The varying degrees of heterogeneity observed in different subgroups highlight the importance of considering specific clinical factors when interpreting the results. Future studies should aim to minimize heterogeneity by using standardized protocols for orthodontic treatment and root resorption assessment. Additionally, further investigation into the potential sources of heterogeneity, such as differences in treatment mechanics and patient-specific factors, is warranted to improve the precision and reliability of meta-analytic findings.

Further subgroup analyses showed significant differences in the impact of various factors on root resorption, such as different measurement methods, whether tooth extraction was performed, and tooth position. Specifically, in study groups using advanced imaging techniques like CBCT, teeth that had undergone root canal therapy exhibited a more pronounced trend of root resorption, whereas studies using traditional panoramic and periodontal X-rays showed less root resorption in treated teeth. This phenomenon may stem from the advantages of CBCT in three-dimensional imaging, allowing for more accurate detection of subtle changes in root resorption, while traditional two-dimensional imaging methods may underestimate the actual extent of root resorption [28]. However, it is important to consider that the enhanced sensitivity of CBCT may also lead to the detection of minor root resorption that is not clinically significant. The high diagnostic sensitivity of CBCT can result in the identification of very small resorption sites that, while statistically detectable, may not necessarily translate into real clinical risk for the patient. In other words, minor root resorption detected by CBCT does not always mean that the tooth is at significant structural risk or will lead to clinical complications such as increased mobility or tooth loss. The challenge lies in distinguishing between clinically significant root resorption that requires intervention and minor resorption that may be a normal variant or a transient finding without long-term consequences. Clinicians must balance the diagnostic advantages of CBCT with the potential overestimation of minor resorption. This requires careful interpretation of CBCT findings in the context of the patient’s overall clinical presentation, including symptoms, tooth mobility, and the presence of other risk factors for root resorption.

In comparisons between extraction and non-extraction treatments, the extraction group showed an increasing trend in root resorption, while the non-extraction group exhibited almost no significant differences. This may be related to the redistribution of orthodontic forces after extraction. The increased spacing between teeth due to extraction may lead to certain teeth bearing greater movement forces, thereby increasing the risk of root resorption [29]. After extraction, orthodontic forces need to be redistributed across a wider dental arch, which may cause some teeth to experience greater tension or pressure, thereby inducing root resorption. Additionally, the movement path and direction of teeth after extraction may change, which could increase pressure on the roots in specific directions, promoting the occurrence of root resorption.

The different positions of the teeth are also important factors affecting root resorption. Our analysis indicated that posterior teeth exhibited a slightly higher trend of resorption after root canal therapy, while anterior teeth showed no significant differences. This may be related to the differing directions and magnitudes of forces applied to anterior and posterior teeth during orthodontic treatment. Anterior teeth typically require more rotation and movement, whereas posterior teeth involve more vertical and lateral movements, which may result in a higher risk of root resorption in posterior teeth [30]. Posterior teeth bear greater loads during chewing, and the forces applied during orthodontic treatment may be more complex and multidirectional, increasing the possibility of root resorption. Moreover, different types of teeth, such as incisors, canines, and molars, have varying root structures and morphologies, which may influence their response to ortho-dontic forces and, consequently, their risk of root resorption [31]. For instance, molars, with their multiple roots, may exhibit different resorption patterns and degrees during orthodontic treatment. The multiple roots of molars may lead to more complex resorption patterns under orthodontic force, whereas single-rooted teeth like incisors may be more prone to uniform root resorption when subjected to force.

Therefore, although this meta-analysis indicates that endodontic treatment alone does not significantly increase the risk of root resorption during orthodontic treatment, it is necessary to clarify potential confounding factors, such as pre-existing root damage that may be caused by endodontic treatment. Endodontic treatment itself involves mechanical preparation of the root canal, which may weaken the dental structure due to microcracks, perforations, or over-preparation. Theoretically, these iatrogenic defects may make the tooth more susceptible to resorption under orthodontic load. To control for this confounding factor, our inclusion criteria required that the integrity of the root be confirmed radiographically before orthodontic treatment. Specifically, studies that reported visible root defects before treatment (such as fractures, external resorption) or used inadequately filled endodontic teeth (such as underfilling, overfilling/underfilling) were excluded. In addition, all included studies used standardized radiographic methods (CBCT, periapical radiographs, or panoramic radio-graphs) to assess root resorption, ensuring the documentation of baseline root structural integrity. However, limitations still exist. Few existing studies clearly report the root condition before endodontic treatment or technical errors during treatment (such as ledges, canal deviation), leading to residual heterogeneity in the analysis. Future studies should establish standardized reporting norms for the quality of endodontic treatment (such as filling density, canal shape) and document pre-treatment defects to more accurately isolate the effects of orthodontic forces. Despite this, this study shows that if the structure of the endodontically treated tooth is intact (using modern endodontic techniques) and the orthodontic force is controlled within the physiological threshold, its resistance to resorption is comparable to that of vital teeth.

The results of this study have important implications for clinical orthodontic treatment. Firstly, root canal therapy itself may not significantly increase the risk of root resorption; however, under specific conditions such as tooth extraction and tooth position, the risk of resorption in treated teeth may increase. Secondly, the choice of imaging methods is crucial for the accurate assessment of root resorption. Although CBCT has significant advantages in detecting root resorption, its high cost and radiation dose limit its widespread use. Therefore, in clinical practice, the accuracy of imaging methods should be weighed against their practical feasibility, and the most suitable assessment method should be selected based on the patient’s specific situation.

This meta-analysis demonstrates that during fixed appliance treatment, root canal-treated teeth do not exhibit significantly different root resorption patterns compared to vital teeth, although additional monitoring may be required in extraction cases due to potentially increased resorption risk. While these findings support the safety of orthodontic treatment in root canal-treated teeth, clinicians should continue to consider individual case factors, including treatment mechanics and duration, when planning orthodontic therapy. Further prospective studies are needed to assess long-term outcomes and specific treatment protocols for this patient population. Additionally, future research should investigate the impact of different root filling materials (e.g. bioceramics vs. gutta-percha) on root resorption risk during orthodontic treatment.

The limitations of current evidence highlight important directions for future research. One notable limitation is that our study was not registered in a prospective database such as PROSPERO. Registration of systematic reviews is considered a best practice as it enhances transparency and reduces the risk of selective reporting and other biases. By not registering our review, we acknowledge that this may introduce potential biases related to the flexibility of our research protocol and the potential for selective outcome reporting. Future systematic reviews should consider registration to improve the rigor and transparency of the research process. Prospective studies with standardized protocols and longer follow-up periods would provide valuable insights into the long-term stability of orthodontically moved endodontically treated teeth. Investigating specific force systems, timing of treatment relative to endodontic therapy, and the impact of root filling materials (e.g. bioceramics vs. gutta-percha) would enhance our understanding of optimal treatment approaches. Integrating advanced imaging technologies with longitudinal clinical studies could provide more precise quantification of root resorption patterns and tissue responses. Additionally, exploring potential biological markers associated with root resorption susceptibility could lead to more pers-onalized treatment plans based on individual risk factors rather than broad categorizations based solely on pulp status.

## Conclusion

This meta-analysis demonstrates that root canal-treated teeth do not exhibit significantly different root resorption patterns compared to vital teeth during fixed orthodontic treatment, though extraction cases may require additional monitoring due to potentially increased resorption risk. While these findings support the safety of orthodontic treatment in endodontically treated teeth, clinicians should continue to consider individual case factors, including treatment mechanics and duration, when planning orthodontic therapy. Further prospective studies are needed to evaluate long-term outcomes and specific treatment protocols for this patient population.

## Declarations

### Ethics approval and consent to participate

An ethics statement is not applicable because this study is based exclusively on published literature.

## Data Availability

All data generated or analyzed during this study are included in this article. Further enquiries can be directed to the corresponding author.
